# Enhancing resistance training adherence in older adults with sarcopenia or osteoporosis: a study on referral success rates

**DOI:** 10.3389/fpubh.2025.1632960

**Published:** 2025-08-18

**Authors:** Ching-Huang Lin, Yun-Ju Cheng, Ching-Ping Hsu, Gwo-Chi Hu, Hsin-Yin Hsu, Yu-Ning Chien, Hsin-Hui Lin, Lee-Ching Hwang, Hsiao-Chi Ma, Fang-An Lin, Meng-Ting Tsou, Tung-Ke Wu

**Affiliations:** ^1^Department of Family Medicine, Taipei MacKay Memorial Hospital, Taipei, Taiwan; ^2^Institute of Long-Term Care, MacKay Medical University, New Taipei, Taiwan; ^3^Department of Medicine, MacKay Medical University, New Taipei, Taiwan; ^4^Department of Rehabilitation, Mackay Memorial Hospital, Taipei, Taiwan; ^5^Department of Health and Welfare, College of City Management, University of Taipei, Taipei, Taiwan; ^6^Independent Researcher (Electrical and Electronics Engineering), National Taiwan University, Taipei, Taiwan

**Keywords:** sarcopenia, osteoporosis, progressive resistance training, patient education, cardiopulmonary exercise testing

## Abstract

**Background:**

Sarcopenia and osteoporosis increase the likelihood of disability and caregiving burden. While progressive resistance training (PRT) is effective in mitigating these outcomes, patients often struggle to find suitable, long-term training facilities, making it difficult to adhere to exercise prescriptions.

**Objectives:**

This feasibility study aimed to familiarize patients with PRT through educational training by a geriatrician, enabling them to identify a suitable long-term community-based training program.

**Participants and methods:**

Forty-one patients diagnosed with osteoporosis or sarcopenia at a medical center in Taiwan were enrolled via the researchers’ LINE app platform. Finally, 11 participants with osteoporosis were recruited. Among them, four had vertebral compression fractures and two also met the diagnostic criteria for sarcopenia. The median age was 68 (range 63–69) years, DXA femoral neck T-score was −3.3 (−3.5–−2.2), and grip strength was 22.4 (20.3–26.7) kg. After cardiopulmonary exercise testing (CPET), participants received up to 10 PRT sessions, with vital signs monitored. In each session, researchers focused on six key learning points of free-weight PRT, addressed difficulties hindering progress, encouraged participants to find community-based training courses, and provided training summaries to external trainers. Descriptive statistics summarized patient data and referral rates. The primary outcome was the success of referrals to community-based training. Secondary outcomes, to be reported later, included changes in grip strength, DXA bone mineral density, DXA muscle mass, CPET results after 6 months, and sustainability of long-term resistance training (RT) in older people with osteoporosis or sarcopenia.

**Results:**

The recruitment success rate was 26.8%. Baseline characteristics did not correlate to successful referrals. A total of 67 physician-guided PRT sessions were conducted for the 11 participants. On average, after 4.5 in-hospital sessions, five participants secured self-paid one-on-one RT in the community, and one joined group training, resulting in a referral success rate of 54.5%.

**Conclusion:**

This feasibility study aimed at achieving successful referral for long-term community-based RT. It provides valuable insights for future research on RT for patients with osteoporosis or sarcopenia, making exercise a sustainable and quantifiable intervention.

## 1 Introduction

Sarcopenia and osteoporosis are major causes of disability among older adults and pose a significant burden on aging societies. In 2019, Europe and Asia updated the diagnostic criteria for sarcopenia, including insufficient muscle strength, low muscle mass, and poor physical performance (1, 2). These patients are at higher risk of falls and hospitalization (2). According to 2018 United States statistics, 26.5% of individuals aged >65 years fell at least once per year (3). Hip fractures due to falls lead to a one-year mortality rate of 18% in women and 11.2% in men, which is markedly higher than the standard mortality rates of 2.8% in women and 3.6% in men without fractures (4). Osteoporosis is a critical factor that leads to fractures from falls.

Currently, no effective medications exist for sarcopenia, which is primarily treated with nutrition and resistance training (RT) (5). Although various drug treatments for osteoporosis are available in Taiwan, only patients with vertebral or hip fractures receive subsidies. Patients with osteoporosis without fractures need to pay for the medications themselves, which results in low medication usage rates in this group (4). The 2018 LIFTMOR study confirmed that high-intensity resistance and impact training (HiRIT) improved bone density and functional performance (6). The International Osteoporosis Foundation recommends that patients with osteoporosis engage in diverse exercise programs, including resistance and balance training (7, 8). Increasing research recognizes the potential of RT to reverse functional decline and alter frailty trajectories (9). The 2025 ICFSR global consensus states that progressive resistance training (PRT) is crucial for maintaining or improving function among older adults, especially those who are frail and have sarcopenia or osteoporosis (10).

The Spanish Vivifrail program grades physical function among older adults from healthy to frail and provides clinically oriented exercise prescriptions (11). From 2021 to January 2025, four studies involving 20–188 participants each showed improved functional status and reduced fall risk among frail older adults using the Vivifrail program (12–15). However, exercise prescription including PRT as a treatment option may be challenging in clinical applications. First, few doctors inquire about RT or prescribe it (16) likely due to the lack of assessment coverage and busy clinical schedules. Second, many variables in RT for sarcopenia require specific prescriptions for different endpoints. A 2024 NEJM study comparing hip replacement surgery with RT found that surgery was more effective for severe hip osteoarthritis (17), but experts noted that the training method was not suitable for this group (18). A 2021 meta-analysis found that high-intensity RT effectively increased bone density in patients with osteoporosis; however, the studies were highly heterogeneous (19). Thus, prescribing RT for patients with sarcopenia or osteoporosis is highly complex.

However, managing type 2 diabetes, which affects 10% of the global population, requires equally complex medical treatments. The American Diabetes Association updates its treatment guidelines annually to optimize healthcare providers’ prescribing capabilities (20). Therefore, we referred to the recommendations made by experts for clinical trials of drugs used to treat sarcopenia (21). By designing research to identify dosing principles for RT, healthcare providers can prescribe suitable RT exercise regimens to treat sarcopenia. Third, long-term adherence data are lacking in resistance-training studies. Most studies range from 12 to 54 weeks (19), and although they yielded positive results, whether participants continue training post-study remains unknown. Finally, patients with sarcopenia and osteoporosis find it difficult to access long-term training programs. This difficulty arises from factors related to the patients, the fitness industry, and medical institutions. The general public is unfamiliar to RT, and older adults believe that walking for an hour each day constitutes sufficient exercise. The cost of a one-on-one training session is $50–$80 per hour, with a weekly session equating to the costs of the physical intervention group in the LIFE study (22) and far exceeding the monthly pension of $125 for older adults aged 65–89 years in Taiwan. Both the fitness industry and medical institutions tend to adopt cautious approaches when dealing with frail, osteoporotic, or sarcopenic older individuals (10).

As osteoporosis and sarcopenia progress with age, long-term RT is required. Exercise referral schemes (ERS) have existed for many years, in which primary care physicians refer individuals with chronic diseases and low activity levels to exercise professionals for training. However, most healthcare systems have not yet established effective models (23). Despite such support schemes, the factors influencing exercise behavior in older adults remain highly complex (24). Older adults often have limited financial resources and reduced physical resilience, resulting in a lower margin for error in adhering to health interventions. These limitations are evident in physical training programs, depicted by the low recruitment and adherence rates. Accordingly, feasibility studies are recommended to enhance the rigor of future trials (25). Therefore, this feasibility trial targeted patients with osteoporosis or sarcopenia who were unable to find suitable training locations or have ineffective training. Coaches with a medical background provide RT and education, conducting up to 10 sessions once weekly over 26 weeks. The primary aim of this study was to evaluate the proportion of patients successfully referred for self-paid community training after receiving RT education. The results are expected to support future research by providing participants with long-term community RT.

## 2 Materials and methods

### 2.1 Study design

**Figure 1:**
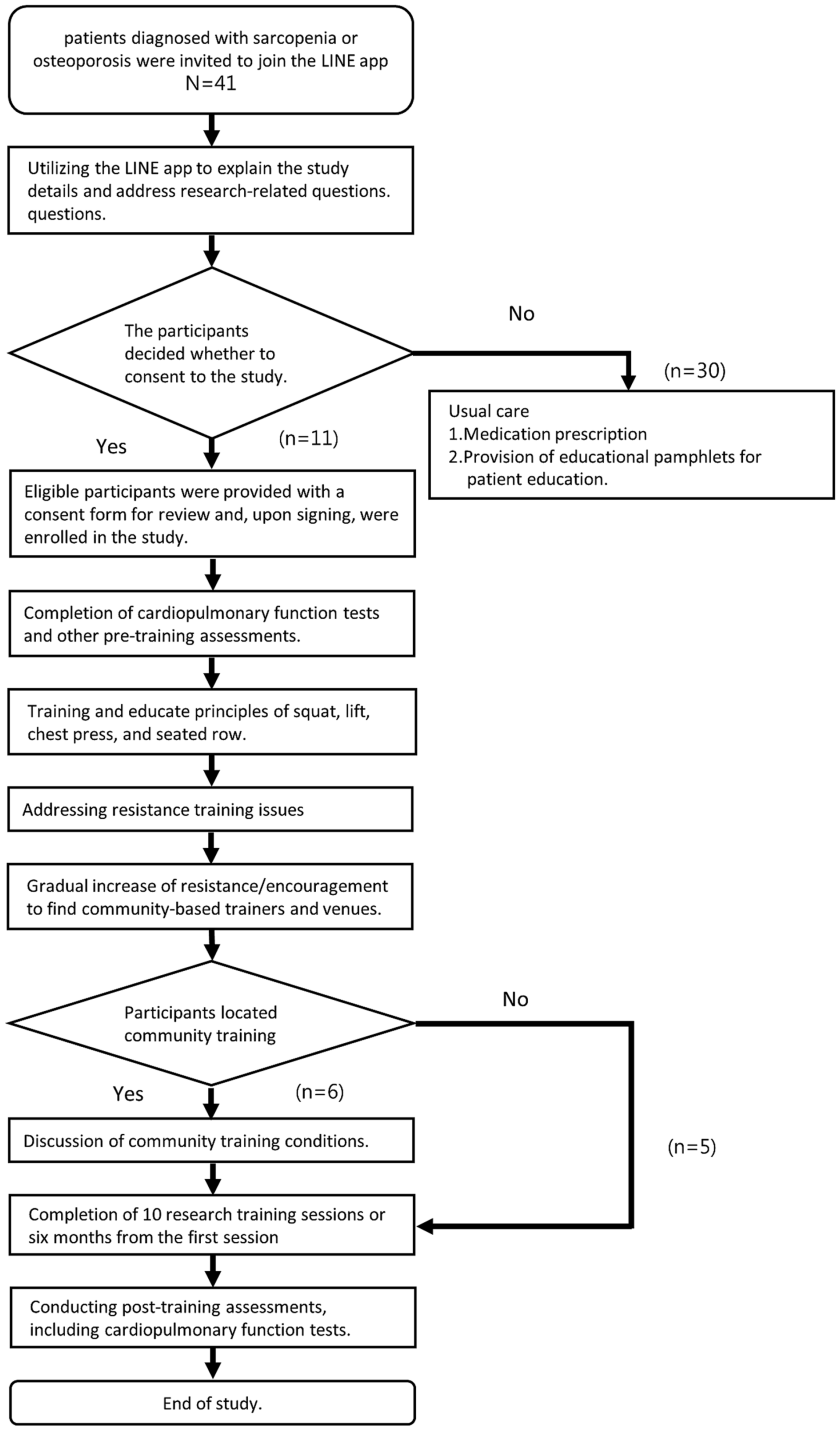
Research flowchart.

This was a single-center feasibility trial offering one-hour, one-on-one, free weight RT sessions weekly, including squats, lifts, pushes, and pulls. The training was adjusted based on the participants’ fitness levels, including sets, repetitions, and rest periods. Researchers conducted up to 10 training and education sessions. Researchers also encouraged participants to find suitable community training locations and coaches using what had been taught about the key points of RT. Researchers may provide recommended locations and coach lists if needed (Figure 1).

### 2.2 Study setting

This study utilized an unused space at Mackay Memorial Hospital equipped with dumbbells, kettlebells, weighted vests, homemade lightweight barbells, resistance bands, and weighted cables. The researchers are certified trainers who provide one-on-one training. When the participants could handle greater resistance, they trained at a nearby gym, covering their entrance fees while receiving free coaching. This study was reviewed and approved by the Mackay Memorial Hospital Institutional Review Board (Approval Number: 23MMHIS223e). All participants provided written informed consent to participate in this study.

### 2.3 Participants and recruitment

Eligible patients from the Family Medicine Clinic at Mackay Memorial Hospital, diagnosed with osteoporosis or sarcopenia according to the 2019 Asian criteria (1, 4), received education on disease consequences and treatment options, such as nutrition and exercise. The recruitment strategy involved three steps (Figure 1). First, doctors emphasized the importance of RT and provided information about the study along with contact details via the Line App. This allowed the research team to invite participants to join the app for personalized one-on-one communication and Q&A. Second, researchers sent out documents and a one-minute training video, which included eight slides outlining the study’s purpose, methods, and expected outcomes. This helped potential participants to understand the training method and encouraged them to attend in-person sessions. Third, for those willing to participate, the research team arranged follow-up visits to assess eligibility.

### 2.4 Inclusion and exclusion criteria

The inclusion criteria for the study were as follows: individuals diagnosed with possible sarcopenia or sarcopenia according to the 2019 Asian criteria (1), or those meeting the diagnostic standards for osteoporosis and osteopenia (4); capable of moving independently; willing to comply with medical prescriptions and undergo pre-exercise evaluation; and willing to find nearby fitness locations and courses.

Osteoporosis was defined as a T-score of ≤ − 2.5 at the femoral neck or total hip, based on the WHO criteria. Sarcopenia was diagnosed according to the 2019 Asian Working Group for Sarcopenia (AWGS) criteria, which require a handgrip strength <28 kg for men and <18 kg for women, along with a height-adjusted appendicular skeletal muscle mass of <7.0 kg/m^2^ for men and <5.4 kg/m^2^ for women. The exclusion criteria comprised clinical judgment indicating difficulty adhering to medical advice (significant cognitive impairment [MMSE ≤20], severe psychiatric disorders, such as schizophrenia, affective disorders: depression, bipolar disorder); severe auditory or visual impairments; inability to perform exercise tests, including cardiopulmonary exercise testing (CPET); terminal illness with life expectancy <12 months; hospitalized within the past 6 months due to acute or subacute health issues (e.g., heart disease, cerebrovascular disease, cancer, arthritis, and fractures) or unstable chronic conditions; systolic blood pressure (BP) ≥ 170 mmHg or diastolic blood pressure ≥100 mmHg (measured twice using the standard method) and resting heart rate >120 bpm; unstable chronic conditions; and cardiac patients with conditions including arrhythmia, or STT depression ≥2 mm, a decrease in SBP during CPET, angina symptoms, dyspnea or dizziness during low-intensity exercise (metabolic equivalent task <4), or internal cardiac defibrillator (ICD) implantation.

Eligible participants reviewed and signed consent forms and appointments were arranged for rehabilitation assessments and CPET. Participants unable to join were respected, to ensure voluntary and informed participation, and to maintain ethical standards and safety.

### 2.5 Sample size

Older individuals in Asian cultures tend to be less receptive to moderate-to-high-intensity RT. Therefore, this study conducted a feasibility test in older individuals with osteoporosis or sarcopenia. We sought to determine if RT and education delivered by certified physicians could improve participation rates in this group. The sample size depended on the actual number of recruits. We recruited participants 3 months before the research funding ended to provide a maximum of 10 training sessions.

### 2.6 Intervention description

#### 2.6.1 Resistance training trainers

This study included three trainers: two doctors with Family Medicine and Geriatric Medicine licenses certified by ACSM-CPT (American College of Sports Medicine certified personal trainer) and NASM-CES (National Academy of Sports Medicine Corrective Exercise Specialist). The third trainer was a computer engineer with “Fitness Instructor Certification Level C” certification, previously involved in developing the ASUS VivoWatch®. This combination was aimed at designing a feasible RT and education program for older individuals with osteoporosis and sarcopenia.

#### 2.6.2 Progressive resistance training content

The goal was to enable participants to find suitable coaches and training venues in their communities for long-term training. The intervention included PRT and education. The participants performed the correct movements and understood the principles of PRT. Four primary exercises, namely deep squats, deadlifts, bench presses, and seated rows, were selected for the study. The primary exercises trained major muscle groups based on ACSM definitions and had the potential to maintain mobility and prevent falls (26, 27).

#### 2.6.3 Resistance training frequency and volume

According to expert recommendations, RT in patients with osteoporosis should be conducted at least twice per week. Owing to manpower and space constraints, this study adopted PRT once a week, with each session lasting for 1 h. Each exercise used an 8–12 repetitions maximum (RM) weight, performing 6–10 repetitions for 3–4 sets (6, 7, 27).

#### 2.6.4 Supervision and key learning points of resistance training movements

Since all participants were diagnosed with osteoporosis, including four with vertebral compression fractures, supervision focused on form and alignment. The participants were guided to maintain a neutral spine and perform hip-hinge movements, and the resistance was increased gradually (7). Considering their older age and higher cardiovascular risk, the Valsalva maneuver was avoided during training (27). Table 1 provides the educational details on the training movements.

**Table 1:** Teaching principles of free-weight resistance training exercise.

Exercise	Therapeutic goal	Description	Regressed or progressed
Squats	Strengthens leg muscles (quadriceps, hamstrings, and glutes) Increases functional mobility for daily activities	Execute feet shoulder-width squats using a weighted cable attached to a belt to minimize additional spine loading and bending. Perform three sets of squats, with each set consisting of 6–10 repetitions. Rest for 2 min between sets.	Chair height: above knee➔ beneath knee Weight progression: body weight➔ adjustable weight vest➔weighted cable➔ barbell
Deadlift	Increases full-body strength, particularly in the posterior chain muscles Strengthens the core, lower back, grip, and postural muscles	Execute a conventional deadlift while maintaining torso stability, ensuring a neutral shoulder girdle and spine position. Perform three sets of deadlifts, with each set consisting of 6–10 repetitions. Rest for 2 min between sets.	Progression: Body weight ➔ Low weight barbell (1 kg aluminum material) + Weight plate up to 10 kg ➔ Weighted cable up to 30 kg ➔ Gym facilities
Seated row	Strengthens upper back muscles, including latissimus dorsi, rhomboids, and trapezius Improves overall upper body strength Strengthens muscles, supporting the spine and shoulders Improves pulling and gripping strength for daily activities	Execute seated row exercise while maintaining upper trunk stability by ensuring a neutral shoulder girdle and spine position during the session to avoid twisting or bending. Avoid momentum-driven movements or excessive torso oscillation. Perform three sets of seated rows, with each set consisting of 6–10 repetitions. Rest for 2 min between sets.	Progression: seated row➔ horizontal pull
Chest press	Hypertrophy and strengthening of the pectoralis major, triceps brachii, and anterior deltoid muscles Augmentation of overall upper extremity muscular strength and endurance	Execute chest press with feet planted firmly on the ground, focusing on a neutral shoulder girdle position during the session. Perform three sets of chest presses, with each set consisting of 6–10 repetitions. Rest for 2 min between sets.	Progression: Elastic band ➔Low weight barbell (1 kg aluminum material) + weight plate up to 10 kg ➔ Weighted cable up to 20 kg

Delayed onset muscle soreness could occur 48–72 h after training, which was expected. It was alleviated with stretching, hydration, and proper rest. However, any pain experienced during the training session was reported to the trainer immediately.

#### 2.6.5 Free weight or machine weight

This study used free weight training. Although machine weight training is simpler for beginners, machines may not suit the body sizes of most participants with osteoporosis or sarcopenia. Free weight training, although more challenging to learn, trains multiple joints and muscle groups, improves balance and muscle strength, and increases safety during daily activities (27). Once learned, various RT tools can be used to follow the exercises prescribed by the doctors. Although the ICFSR guidelines suggest that high-intensity training can be introduced without delay for frail older individuals by starting with a 1 RM assessment (10), the participants in this study had a median lumbar spine bone density of 0.674 g/cm^2^, with a T-score of −3.4. Therefore, training started with body weight, and participants’ movements were observed closely and adjusted before progressing to low-load weights, including dumbbells, weighted vests, elastic bands, or custom lightweight barbells developed by the researchers: aluminum barbells and wooden deadlift racks. Each time, the initial training weight was approximately 30% lower than the maximum weight. Each exercise was performed in 6–10 repetitions and 3–4 sets, with each set increasing the weight by 1–2 kg. When the resistance exceeded 10 kg, the weighted cable training equipment BH P1 SE® was used. Notably, different tools were sometimes used within the same exercise session—for example, dumbbells for the first two sets and BH P1 SE® for the final two. As training load increased, sessions were moved to the gym.

#### 2.6.6 Teaching principles of progressive resistance training

To enable participants to find PRT facilities in their communities, the teaching focused on six points. First, correct posture was emphasized (Table 1). If the participant agreed, a synchronous recording was conducted during the training to allow the participant to watch and learn. Second, for training aimed at increasing muscle strength, the recommended training volume was 1–3 sets per exercise. Third, training intensity involved selecting resistance that could be repeated 8–12 times; if the resistance could be repeated more than 15 times, it was considered light and was increased. Fourth, resting for 2–3 min between sets was recommended, with longer rest for heavier resistance or when training large muscle groups with multiple joints (27). Fifth, post-training protein supplementation and stretching exercises were recommended to help relax the muscles. Sixth, delayed onset muscle soreness could occur 48–72 h after training. It was alleviated with stretching, hydration, and proper rest. However, any pain experienced during the training session was reported to the trainer immediately.

### 2.7 Monitoring training condition and referral success rate

#### 2.7.1 Vital signs

To ensure the safety and effectiveness of the training process, body weight and grip strength were measured before each session, as well BP and pulse before, during, and after training.

#### 2.7.2 Pain and movement limitation

If improper movements or pain occurred during training, the exercise movements and intensity were adjusted (Table 1). In case the situation did not improve, other professionals such as Pilates instructors, physical therapists, neurosurgeons, and rehabilitation physicians were consulted for assistance (see Section 3.3 for a detailed description).

#### 2.7.3 Training volume

During each session, the training volume (training weights and repetitions) was recorded.

### 2.8 Referrals to community training facilities and follow-up

Participants were encouraged to identify venues and coaches in the community offering similar training. In case after the fourth training session and a community training venue had not been found, the researchers inquired about any difficulties encountered. If necessary, the researchers provided a list of recommended community trainers, participated in trial sessions to observe interactions, and evaluated the potential for long-term training. If both parties wished to continue training with the participant’s consent, the researchers provided the community trainer with the participant’s training status, including load capacity, specific movements requiring attention, BP, and heart rate during training. Arrangements were made between community and in-hospital training to track the effectiveness of community training. There were no conflicts of interest between the researchers and community trainers.

#### 2.8.1 Safety monitoring

Within 2 days post-training, participants were contacted via the Line App to inquire about muscle soreness or discomfort. If no issues arose during the first two community training sessions, no further inquiries were made. The pain was assessed and addressed accordingly.

#### 2.8.2 Data collection

Anthropometric measurements (height, weight, and calf circumference), grip strength, five-time chair stand test, and CPET were performed before training. Questionnaires included the SARC-F, Fried phenotype, geriatric depression scale (GDS), and instrumental activities of daily living (IADL) scale.

Participants self-paid for dual-energy X-ray absorptiometry (DXA) scans for bone mineral density (BMD) and muscle mass estimations. The BMD of the femoral neck, total hip, upper 4 lumbar vertebrae were evaluated by DXA (Horizon A®, Hologic Inc., Danbury, CT, United States), and the T-scores were obtained. A T-score of −2.5 or lower in any of these areas met the WHO criteria of osteoporosis. Appendicular lean mass was determined using whole-body DXA. Each participant underwent measurements using the same DXA at baseline and follow-up. Grip strength was measured using a hydraulic-type dynamometer (Jamar, seated with the elbow bent at 90 °) or a spring-type dynamometer (CAMRY, standing with the arm fully extended). Both hands or the dominant hand were tested at least twice, and the highest reading was recorded. AWGS 2019 definition of sarcopenia includes height-adjusted muscle mass: <7.0 kg/m^2^ in men and <5.4 kg/m^2^ in women and handgrip strength <28 kg for men and <18 kg (1).

### 2.9 Outcome measures

The primary outcome was the proportion of successful referrals for long-term community training after the intervention. Secondary outcomes included changes in grip strength, DXA bone mineral density, DXA muscle mass, and CPET cardiopulmonary function test after 6 months of training.

### 2.10 Statistical analysis

Baseline characteristics of the participants are presented using descriptive statistics, with medians used to show central tendencies. The Wilcoxon rank-sum test was used to compare the medians of the two independent populations. The Fisher’s exact test was used to test the differences in categorical variables. All statistical outcomes were examined against a *p*-value of 0.05 to determine statistical significance. Statistical analyses were performed using STATA 16.1 software.

## 3 Results

### 3.1 Participant characteristics

The first participant in this study completed enrollment on 8 January 2024 and began the first in-hospital RT provided by the researchers on 8 March 2024. By 8 January 2025, 41 potential participants were invited to join the LINE app, and 11 participants were enrolled. The recruitment success rate was 26.8%. All enrolled participants met the diagnostic criteria for osteoporosis, with four having vertebral compression fractures and two meeting the diagnostic criteria for sarcopenia.

The median age of the 11 participants was 68 (63–69) years, with a median BMI of 21.2 (20.7, 23.9) kg/m^2^, and a T-score of −3.4 (−4.1, −3.2). The median DXA bone mineral density was 0.679 (0.67, 0.76) g/cm^2^ for the lumbar spine, 0.501 (0.46, 0.54) g/cm^2^ for the femoral neck. The appendicular skeletal muscle mass index (ASMI) was 5.55 (5.3–5.9), whereas the median grip strength was 22.4 (20.3–26.7) kg. The median time for the 5-time chair stand test was 8.0 (6.5–10.1) s. For cardiopulmonary function, the medians peak oxygen uptake was 24.9 (18.9–25.2) mL/kg/min, with participants achieving a median of 87% (67.5–94.0) of the predicted peak oxygen uptake. The median peak workload was 110 (75–127) W (Table 2).

**Table 2:** Participant baseline characteristics (*N* = 11).

Case	A	B	C	D	E	F	G	H	I	J	K	Median (Q1, Q3)
Success Referral	US	US	SR	SR	US	SR	US	SR	SR	US	SR	
Age	76	67	68	69	68	63	66	57	73	54	68	68 (63, 69)
Body height (cm)	145.0	149.0	163.0	146.0	156.0	163.0	151.0	158.0	155.0	158.0	154.0	155 (150, 158)
Body weight (Kg)	44.1	45.8	56.4	46.4	51.6	63.4	61.4	54	35.1	61.5	49	51.6 (46.1, 58.9)
BMI (Kg/m^2^)	21	20.6	21.2	21.8	21.2	23.9	26.9	21.6	14.6	24.6	20.7	21.2 (20.9, 22.9)
Osteoporosis	Yes	Yes	Yes	Yes	Yes	Yes	Yes	Yes	Yes	Yes	Yes	Yes
T-score (overall)	−3.8	−4.5	−3.3	−3.3	−4.4	−3.1	−3.1	−3.5	−4.5	−3.4	−2.6	−3.4(−4.1, −3.2)
Lumbar T	−1.3	−4.5	−3.3	−3.3	−4.4	−3.1	−2.7	−3.5	−3.4	−3.4	−2.6	−3.3(−3.5, −2.9)
Femoral neck T	−3.8	−3.5	−2.6	−3.1	−3.4	−2.2	−3.1	−3	−4.5	−3.4	−2.5	−3.1 (−3.5, −2.8)
BMD (g/cm^2^)												
Lumbar	0.9	0.553	0.779	0.679	0.562	0.705	0.754	0.667	0.669	0.669	0.756	0.679 (0.67, 0.76)
Femoral neck	0.429	0.463	0.672	0.501	0.466	0.606	0.51	0.511	0.348	0.472	0.57	0.501 (0.46, 0.54)
Compression Fracture	T12, L2	No	No	L2	No	No	T11, T12	No	No	No	T11	
Sarcopenia (AWGS 2019)	No	Yes	No	No	No	No	No	No	Yes	No	No	
Grip strength (Kg)	26.7	17.1	24.4	21.6	20.3	27.5	23.0	29.0	9.8	22.4	20.3	22.4 (20.3, 25.6)
ASMI	6.05	4.20	5.46	5.21	6.43	5.70	6.74	5.55	3.57	5.53	5.59	5.55 (5.3, 5.9)
Muscle mass (g)												
Left arm	1868	1,084	1,632	1,291	1,580	1,464	1,584	1,553	1,047	1,545	1,473	1545^a^
Right arm	1,632	1,081	1838	1,239	1,665	1927	1889	1,637	1,047	1,432	1,563	1632^b^
Left leg	4,568	3,651	5,545	4,141	6,049	5,603	5,818	5,348	2,813	5,233	4,919	5233^c^
Right leg	4,646	3,621	5,488	4,434	6,349	6,141	6,066	5,308	3,122	5,122	5,292	5292^d^
Trunk	14,894	23,943	36,979	28,729	35,121	38,216	38,678	15,817	22,199	32,309	32,806	32309^e^
Calf circumference (cm)												
Left	29.5	30.7	32.6	34.0	35.0	35.6	34.0	32.0	26.0	34.0	33.5	33.5 (31.4, 34)
Right	30.0	31.5	32.8	34.0	35.0	36.6	34.0	32.0	26.0	34.0	33.9	33.9 (31.8, 34)
Regular exercise	No	No	No	No	No	No	Yes	No	No	Yes	No	
SARC F questionnaire	1	0	0	1	1	1	0	0	2	0	0	0 (0, 1)
5-time chair stand test(sec)	6.0	9.8	7.0	5.6	8.0	11.9	8.0	10.4	12.1	6.0	8.2	8.0 (6.5, 10.1)
Fried frailty score	0	3	0	2	0	0	0	0	1	1	1	0 (0, 1)
GDS 5	2	0	1	2	1	1	1	0	1	1	0	1 (0, 1)
IADL	24	21	23	23	21	21	24	24	22	24	24	23 (22, 24)
CPET												
MET	7.9	5.2	7.2	4.8	7.2	6.7	7.1	7.1	4.9	7.7	5.7	7.1 (5.5, 7.2)
Peak oxygen uptake, mL/kg/min	27.6	18.1	25.2	16.8	25.2	23.4	24.9	25.0	17.1	27.1	19.8	24.9 (18.9, 25.2)
Predicted peak O_2_ uptake achieved (%)	111	68	95	64	95	85	93	87	67	92	61	87 (67.5, 94)
Peak workload, W	112	43	105	51	110	150	143	99	32	150	111	110 (75, 127)

IADL, Instrumental Activities of Daily Living; ASMI, Appendicular skeletal muscle mass index; CPET, Cardiopulmonary exercise testing; GDS, Geriatric depression scale; MET, metabolic equivalent; SR, Successful Referrals; UR, Unsuccessful Referrals. a (1,377, 1,568); b (133, 1752); c (4,355, 5,574); d (4,540, 5,777); e (23,071, 36,050).

Among the 11 participants, nine had no prior experience with physical training. Two participants had a history of physical training, with one having engaged in one-on-one coaching for a year and the other in group hydraulic exercise courses. However, their bone density did not improve, as evidenced by a T-scores of −3.1 (case G) and −3.4 (case J). Hence, they were invited to participate in the study.

### 3.2 Successful referral rates following educational progressive resistance training

From 8 January 2024 to 8 January 2025, 67 RT sessions were provided, consisting of 55 in-hospital and 12 off-site gym sessions. Among the 11 participants, six found long-term training locations in the community after joining the study. The overall referral success rate was 54.5%. Five participants joined one-on-one courses, and one joined a group course. Among the six participants, four (66.7%) found suitable coaches from the list provided by the researchers. After two to seven in-hospital training sessions, community training courses were conducted, with an average of 4.5 in-hospital training sessions. The average time from the first study training to the first offsite training session was 39 days. Two participants did not participate in the community RT courses due to economic reasons. One participant attended once but did not continue for personal reasons, although they continued to inquire about health-related issues using the Line App. Two participants continued with their original exercise methods (Table 3).

**Table 3:** Initial training conditions and challenges during training.

Case	A	B	C	D	E	F	G	H	I	J	K
SR or UR	UR	UR	SR	SR	UR	SR	UR	SR	SR	UR	SR
Maximum load (Kg) excluding body weight during the first study training session											
Squats	0^#^	8	4	0^#^	0^#^	4	8	0^#^	3	8	20
Deadlift	0^#^	5	0^#^	0^#^	0^#^	10	8	0^#^	0^#^	8	20
Maximum load (Kg) before the first community training session in SR group; or Maximum load in the last study training session in UR group											
Squats	20	8	12	8	24	20	27.5	10	14	37.6	30
Deadlift	22	5	9	7	28	20	27.5	6	3	30	30
First study training session training volume (training weights(kg)*repetitions)											
Squats	0	40^a^	72^c^	0	0	96^d^	80^d^	0	50^e^	240^d^	520^e^
Deadlift	0	30^b^	0	0	0	26^d^	80^d^	0	0	240^d^	500^e^
Last in-hospital training volume(kg) ^†^											
Squats	378^a^^,^^c^	40^a^	164^a^^,^^d^	210^a^	576^e^	370^e^	737.5^f^	230^d^^,^^f^	336^e^	725^g^	760^e^
Deadlift	400^a^^,^^d^	30^b^	60^a^	80^a^	720^e^	340^e^	950^f^	36^d^	50^e^	575^f^	780^e^
Problem encountered during training sessions (solution)											
Unable to perform hip hinge properly (Try using different commands to guide exercise movement)											
Case	■							■	■		
Low back discomfort or limited shoulder range of motion when training (Refer to Pilates, neurosurgery, or rehabilitation, respectively)											
Case				■		■				■	
Hard to maintain proper breathing during the workout (Refer to Physical therapist)											
Case					■						
First training date in study and in community; sessions and interval between 1st study training and 1st community training											
In study	2024/3/8	2024/5/17	2024/7/12	2024/7/12	2024/7/12	2024/9/12	2024/9/4	2024/9/4	2024/9/26	2024/10/23	2024/10/18
Community			2024/8/22	2024/9/10		2024/9/23		2024/9/21	2024/11/13		2024/12/13
Training session (N)			4	7		2		2	6		5
Time spent (days)			41	60		11		17	48		56
Average blood pressure and heart rate during training (mmHg, BPM)											
Before training	125/71, 68	128/73, 72	131/79, 69	116/61, 62	115/68, 83	116/73, 61	145/82, 90	123/57, 97	122/73, 81	137/79, 67	107/63, 62
After squats	133/72, 67	141/81, 80	162/85, 72	119/69, 66	122/73, 83	135/78, 60	178/89, 100	127/73, 104	129/71, 79	136/79, 88	122/70, 62
After lifts	133/71, 67	143/83, 73	168/89, 71	123/67, 69	117/71, 83	138/85, 83	170/89, 96	122/754, 92	122/69, 83	130/71, 76	124/74, 63

SR, successful referral; UR, unsuccessful referral; BP, blood pressure; HR, heart rate.

† Before the first community training session in SR group (Kg); or in the last study training session in UR.

# body weight training.

a custom lightweight barbells.

b Elastic band.

c Weighted vest.

d Dumbbell or kettlebell.

e Fitness board device.

f Barbell or Smith machine.

g Leg press machine.

Age, BMI, T-scores of the lumbar spine and femoral neck obtained from DXA, appendicular muscle mass, physical performance, cardiopulmonary function, changes in blood pressure and heart rate before and after training, changes in load-bearing capacity, presence of vertebral compression fractures, regular exercise habits, mood, activities of daily living, frailty status, and other variables were not related to the success of referral to community training (Table 4).

**Table 4:** Baseline and post-training differences between unsuccessful and successful referral groups.

	Median (Q1, Q3)/ (n, %)	Unsuccessful referrals	Successful referrals	*p*-value
		*N* = 5	*N* = 6	
Age	Median (Q1, Q3)	67 (66,68)	68 (63,69)	0.7125
BMI		21.20 (21,24.6)	21.40 (20.7,21.8)	0.6473
T-score				
Lumbar		−3.40 (−4.4, −2.7)	−3.30 (−3.4, −3.1)	0.6466
Femoral neck		−3.40 (−3.5, −3.4)	−2.80 (−3.1, −2.5)	1.000
ASMI (Kg/m^2^)		6.05 (5.53,6.43)	5.51 (5.21,5.59)	0.2012
Grip strength (Kg)		22.40 (20.3,23)	23.00 (20.3,27.5)	0.6473
Five-time chair stand test (sec)		8.00 (6,8)	9.30 (7,11.9)	0.2711
Peak workload, W		112 (110,143)	102 (51,111)	0.1432
MET		7.2 (7.1,7.7)	6.2 (4.9,7.1)	0.0988
SBP				
Initial		128 (125,137)	119 (116,123)	0.1432
After squats		136 (133,141)	128 (122,135)	0.2343
After deadlift		133 (130,143)	123.50 (122,138)	0.4642
HR				
Initial		72 (68,83)	65.50 (62,81)	0.3602
After squats		83 (80,88)	69 (62,104)	0.1441
After deadlift		76 (73,83)	77 (69,83)	0.7125
Squats weight (Kg)				
Initial		8 (0,8)	3.5 (0,4)	0.7766
Post training		24 (20, 27.5)^a^	13 (10,20)^b^	0.3130
Lift weight (Kg)				
Initial		5 (0, 8)	0 (0,10)	0.9205
Post training		27.5 (22, 28)^a^	8 (6,20)^b^	0.2722
Squats training volume (Kg)				
Initial		40 (0,80)	61 (0, 96)	0.7792
Post training		576 (378, 725)^a^	283 (210, 370)^b^	0.4286
Lift training volume (Kg)				
Initial		30 (0,80)	0 (0, 26)	0.4859
Post training		575 (400, 720)^a^	70 (50, 340)^b^	0.3290
Compression Fracture	Y (n, %)	2 (40.00)	2 (33.33)	1.000
	N	3 (60.00)	4 (66.67)	
Regular exercise	Y	2 (40.00)	0 (0.00)	0.1820
	N	3 (60.00)	6 (100.00)	
SARC F questionnaire	0	3 (60%)	3 (50%)	1.000
	1–2	2 (40%)	3 (50%)	
GDS5	0	1 (20%)	2 (33.33%)	1.000
	1–2	4 (80%)	4 (66.67%)	
IADL	21–22	2 (40%)	2 (33.33%)	1.000
	23–24	3 (60%)	4 (66.67%)	
Fried frailty score	0	3 (60%)	3 (50%)	1.000
	1–5	2 (40%)	3 (50%)	

BMI, body mass index; IADL, Instrumental Activities of Daily Living; ASMI, Appendicular skeletal muscle mass index; GDS, Geriatric depression scale; HR, heart rate; MET, metabolic equivalent; SBP, systolic blood pressure.

a Maximum load in the last study training session in Unsuccessful Referral group.

b Maximum load before the first community training session in Successful Referral group.

### 3.3 Educational resistance training and problem solving

This study introduced RT to the participants (Table 1), enabling them to find long-term training locations in the community. As some studies have indicated, older adults are relatively unfamiliar with RT (28). The participants in this study also found RT relatively unfamiliar; however, with one-on-one guidance from physicians, they were able to overcome safety concerns and gradually increase training resistance. Four participants (cases D, E, F, and J) encountered significant challenges that required additional professional intervention (Table 3).

For participants with significant osteoporosis or sarcopenia, discomfort during training may lead to abandonment of RT. This feasibility study, designed with education and training by coaches with medical backgrounds, helps overcome training obstacles and increases the likelihood of adherence to long-term RT.

### 3.4 Blood pressure change during training

Increased BP was observed in two of the 11 participants (Cases C and G) during the rest period between movements. While the chronic effects of RT on reducing BP are well established (29), BP may increase immediately after exercise, although this increase is typically not sustained after a few minutes (30). Emphasizing proper breathing techniques during both the concentric and eccentric phases of movement and providing guidance to avoid the Valsalva maneuver are essential to mitigate the risk of adverse vascular events (31). Extended BP monitoring, such as home BP tracking, was considered if no acute discomfort was reported immediately after training.

### 3.5 Weight capacity improvements in the study and community

Various resistance training tools were incorporated into the physician-led training, including custom lightweight barbells, elastic bands, weighted vests, dumbbells or kettlebells, fitness board devices, barbells or Smith machines, and leg press machines. Training volume (resistance × repetitions) increased substantially among participants. In the study phase, squat training volume increased from 61 kg to 283 kg in six participants who were successfully referred to community training. Among the five participants who were not successfully referred, training volume increased from 40 kg to 576 kg (Table 4). Despite receiving the same training protocol during the study, participants referred to community programs engaged in diverse training methods. For example, squat techniques varied and included full, half, quarter, and machine squat. These variations complicate the evaluation of weight capacity improvements during community-based RT. This highlights the need for a resistance evaluation model for older individuals with osteoporosis or sarcopenia rather than standardized movement patterns, considering the significant individual differences in joint mobility and muscle strength among older adults. Standardizing movement patterns may reduce their chances of participating in long-term RT.

## 4 Discussion

This feasibility study was designed to successfully refer patients with osteoporosis or sarcopenia to long-term community RT with several important characteristics. First, the study included patients with bone density T-scores ≦ − 4, a group often excluded from other studies (32–34). However, this study included patients with a median bone density T-score of −3.58 as these patients were at high risk and in greatest need of weight-bearing training. For patients with severe osteoporosis or sarcopenia, finding suitable RT coaches and venues is challenging. Coaches may refuse to train older patients with T-scores ≦ − 3 due to concerns of training-induced fractures. Alternatively, they might opt for low-resistance training, which is safer but potentially less effective in achieving desired outcomes. This study provided both treatment and education to help these high-risk older individuals find suitable locations for long-term training. Second, PRT was provided by certified physicians in medical institutions, where physical responses (e.g., changes in blood pressure, heart rate, and skeletal muscle pain) were closely monitored. This approach helped mitigate concerns about discomfort and injury. Six of the 11 participants were successfully referred to community-based RT programs after completing 2 to 7 in-hospital training sessions (Table 3). After a median of 4.5 sessions, more than 50% of the participants were able to identify suitable coaches and facilities, enabling continued RT to enhance muscle strength and physical function, potentially reducing the risk of falls and disability. Third, the study involved a diverse group of coaches with varying backgrounds. For patients with severe osteoporosis, including those with compression fractures, selecting appropriate exercises to perform and determining resistance levels is complex. Ongoing research is being conducted to identify appropriate training methods for this population (35, 36). Owing to budget constraints, the research team, comprising two doctors and one computer engineer, took on roles beyond physical training, including monitoring participants’ vital signs, addressing training barriers, and developing methods for recording training data. Fourth, cardiopulmonary function tests were conducted prior to the training to observe the participants’ physical condition and BP changes under stress, allowing the develop of tailored training plans. These tests also help quantify physical changes before and after training. In particular, participants with sarcopenia were specifically advised to increase their aerobic exercises, such as yoga, prior to the test. Last, the study selected four multi-joint exercises using moderate resistance, with each exercise repeated 6–10 times in three to four sets. Preliminary results indicated that this approach was safe, effective, and consistent with other research findings (35). More than 50% of participants successfully transitioned to continuous community training and exhibited high acceptance of the program.

### 4.1 Study impact

Successfully referring patients to long-term community training is feasible and helps improve muscle strength and physical performance in the older adult population. The community coach list provided in this study enabled participants to find reliable coaches and enhanced training sustainability. Although the participants could not afford the future gym fees, they can maintain muscle strength using dumbbells, kettlebells, or body weight, thereby delaying the onset of sarcopenia. Elastic bands are easily accessible resistance-training tools; however, sufficient resistance is required to produce training effects (37). All participants learned how to progressively increase resistance and gradually felt their strength improving in daily life. Although training under coach supervision is recommended to avoid inadequate self-monitoring (6), guiding the older adults with correct RT knowledge and methods to increase resistance makes self-training effective.

### 4.2 Study limitations

This study has certain limitations. First, this study did not include male participants, and thus the results cannot be generalized to all patients with osteoporosis or sarcopenia. The primary reasons for this are financial and manpower constraints, as the study was conducted at a single center. Additionally, men generally believe that they can manage physical training independently, resulting in lower willingness to participate in the study. Therefore, the next phase will involve collaboration with more medical institutions to increase the sample size and male participants. Second, this study used a RT protocol of 8–12 repetitions maximum (RM) in 6–10 sets. Unlike most other RT studies that prescribe training load as a percentage of 1 RM, our approach was chosen as regular 1 RM measurement required more manpower and imposed considerable physical load on this cohort without yielding better training outcomes (38). Third, the study was approved by the Institutional Review Board of MacKay Memorial Hospital in July 2023, for the period between November 13, 2023, and November 12, 2024. Participant enrollment occurred between January 8 and October 21, 2024. A longer study period might have improved recruitment but would have required additional resources. Effective RT for osteoporosis and sarcopenia requires substantial time and professional supervision. Fourth, while both conditions benefit from PRT (5, 6, 10), this study aimed to evaluate whether PRT education could promote long-term adherence to appropriately dosed, community-based programs. These are distinct conditions, and ideally, separate analyses would help clarify the factors influencing participation in each group. Due to limitations in staffing, funding, and time, this pilot enrolled 11 older adults with osteoporosis, including two who also had sarcopenia. These preliminary findings will guide future studies with larger samples and separate subgroup analyses. Lastly, this study did not comprehensively record nutritional status, exercise habits, or polypharmacy. According to the recommendations of the European Society for Clinical and Economic Aspects of Osteoporosis, Osteoarthritis, and Musculoskeletal Diseases (ESCEO), a thorough assessment of these factors is essential when evaluating the efficacy of RT for sarcopenia (21). Future research should consider these factors to ensure that they are evenly distributed between experimental and control groups.

### 4.3 Future research suggestions

This study demonstrated the feasibility of educating and referring patients with osteoporosis or sarcopenia to community-based RT following brief, physician-led instruction. Although such sessions are costly, they provide safety advantages. After a median of 4.5 sessions, more than half of the participants were able to access appropriate RT facilities. A time-limited model (e.g., ≤8 sessions over 2 months) may offer a practical solution in aging societies with rising healthcare costs. However, the benefits of longer interventions warrant further evaluation. Future research should explore scalable models to support RT implementation, including: 1. Subsidized training programs. Research grants could cover 80% of training costs, with older participants contributing the remaining 20%. 2. Integration into resident training curricula. Incorporating RT into medical residency programs to foster synergy between clinical care and exercise interventions. 3. Service-learning (S-L) programs. Service-learning could serve as a valuable resource to promote public health education and social engagement among students in health sciences (39) (e.g., nursing or physiotherapy students). Research indicates that nutrition may support muscle synthesis (40–42), and RT can slow the progression of frailty and disability, potentially reducing the financial burden on national health insurance (43). Nutritional supplementation and RT are burdensome for most older adults. Therefore, more research and public education are needed to determine the optimal allocation of limited resources to achieve healthy aging and reduce healthcare expenditure in an aging society.

## Data Availability

The original contributions presented in the study are included in the article/supplementary material, further inquiries can be directed to the corresponding author.
